# T-bet Expression by Foxp3^+^ T Regulatory Cells is Not Essential for Their Suppressive Function in CNS Autoimmune Disease or Colitis

**DOI:** 10.3389/fimmu.2015.00069

**Published:** 2015-02-18

**Authors:** Rhoanne C. McPherson, Darryl G. Turner, Iris Mair, Richard A. O’Connor, Stephen M. Anderton

**Affiliations:** ^1^MRC Centre for Inflammation Research, Centre for Multiple Sclerosis Research and Centre for Immunity Infection and Evolution, University of Edinburgh, Edinburgh, UK

**Keywords:** Foxp3, T-bet, EAE, colitis, autoimmune disease

## Abstract

Accumulation of T regulatory (Treg) cells within the central nervous system (CNS) during experimental autoimmune encephalomyelitis (EAE) is essential for the resolution of disease. CNS Treg cells have been shown to uniformly express the Th1-associated molecules, T-bet and CXCR3. Here, we report that the expression of T-bet is not required for the function of these Treg within the CNS. Using mice that lacked T-bet expression specifically within the Treg compartment, we demonstrate that there was no deficit in Treg recruitment into the CNS during EAE and no difference in the resolution of disease compared to control mice. T-bet deficiency did not impact on the *in vitro* suppressive capacity of Treg. Transfer of T-bet-deficient Treg was able to suppress clinical signs of either EAE or colitis. These observations demonstrate that, although Treg can acquire characteristics associated with pathogenic T effector cells, this process is not necessarily required for their suppressive capacity and the resolution of autoimmune inflammation.

## Introduction

The ability of Foxp3^+^ T regulatory (Treg) cells to acquire prototypic T effector (Teff) cell characteristics has been reported to aid their migration to sites of inflammation and increase their capacity to suppress distinct types of inflammatory response. The expression of IRF4 has been implicated in the control of Th2 responses (1). Induction of STAT3 signaling in Treg, resulting in expression of RORγt and CCR6, has been identified as a requirement for Treg to suppress Th17 responses (2). Furthermore, CCR6-deficient Treg were inhibited in their ability to migrate to sites of inflammation containing Th17 cells (3–5). Similarly, during Th1-dominated responses, Treg have been shown to acquire expression of the Th1 markers T-bet and CXCR3 (6–8), and CXCR3-deficient Treg are thought to have a reduced capacity for trafficking to sites of Th1 inflammation (9, 10). Indeed, CXCR3-transfected Treg were found to accumulate at sites of inflammation more efficiently than non-transfected Treg in a murine graft versus host model (11). T-bet-deficient Treg have also proved inferior compared to wild-type (WT) Treg at suppressing Th1-led multi-organ autoimmunity in *scurfy* mice (7).

Using experimental autoimmune encephalomyelitis (EAE) as a model of central nervous system (CNS) autoimmune inflammation, we have previously shown that Treg cells accumulate within the CNS during EAE and are essential for resolution of disease (12, 13). Although EAE pathology is considered to be mediated by a heterogeneous mix of Teff cell populations, namely Th1 and Th17 cells (14, 15), the Treg cells that accumulate within the CNS during EAE uniformly express the Th1-associated transcription factor T-bet and the homing molecule CXCR3 (16). In addition to this observation, it has previously been reported that EAE in CXCR3-deficient mice results in a more severe or protracted disease course and reduced accumulation of Treg within the CNS (17, 18). These findings implied that the acquisition of Th1 characteristics by Treg cells might be required for their suppression of CNS inflammation during EAE. Here, we tested this possibility by the generation of mice that lacked the ability to express T-bet specifically in the Treg compartment. We found no evidence that these mice had impaired Treg cell accumulation in the CNS or natural recovery from EAE. Furthermore, therapeutically administered T-bet-deficient Treg cells were able to prevent the development of either EAE or colitis.

## Materials and Methods

### Mice, antigens, and tissue culture medium

Wild-type C57BL/6 (CD45.2/CD90.2 or CD45.2/CD90.1), B10.PLxC57BL/6 (CD45.2/CD90.2), and *Tbx21^-/-^* (KO; obtained from The Jackson Laboratory) were used. Tg4 mice (CD90.1) express a transgenic TCR recognizing the Ac1-9 peptide of myelin basic protein (MBP) (19). Foxp3^tm4(YFP/cre)Ayr^ mice (20) were kindly provided by Dr. A. Rudensky. T-bet^fl/fl^ (21) (kindly provided by Dr. S. Reiner) were crossed with Foxp3^tm4(YFP/cre)Ayr^ mice to generate T-bet^fl/fl^Foxp3-Cre conditional KO (cKO) mice and T-bet^fl/WT^Foxp3-Cre conditional heterozygous (cHet) mice (both CD90.2). These lines expressed YFP under control of the Foxp3 promoter. *Tbx21^-/-^* mice were crossed with Tg4xFoxp3.LuciDTR-4 (CD45.1) (Tg4.WT) (22) to generate *Tbx21^-/-^*Tg4xFoxp3.LuciDTR-4 (CD45.1) (Tg4.T-bet*^-/-^*) mice. Both the Tg4.WT and Tg4.T-bet*^-/-^* lines expressed GFP under control of the Foxp3 promoter. All mice were bred under specific pathogen-free conditions at the University of Edinburgh. All experiments were approved by the University of Edinburgh Ethical Review Committee and were performed in accordance with UK legislation. The myelin oligodendrocyte glycoprotein 35–55 peptide (pMOG) and the MBP Ac1–9 and Ac1–9 (4Tyr) peptides were obtained from Cambridge Research Biochemicals (Cleveland, UK). Tissue culture medium (RPMI 1640 medium) was supplemented with 2 mM l-glutamine, 100 U/ml penicillin, 100 μg/ml streptomycin, and 5 × 10^-5^ M 2-ME (all from Invitrogen Life Technologies, Paisley, UK) plus 10% FCS (Sigma, Poole, UK). Cells isolated from immunized mice were cultured in X-VIVO15^TM^ serum-free medium (Lonza, Walkersville, MD, USA) supplemented with 2 mM l-glutamine and 5 × 10^-5^ M 2-ME.

### Cell purification and culture

CD4^+^ T cells were purified by magnetic cell sorting (Miltenyi Biotec, Germany) prior to surface staining and sorting by FACS. CD4^+^CD62L^hi^CD25^+^GFP^+^ nTreg were isolated from Tg4.WT or Tg4.T-bet^-/-^ mice. CD4^+^CD62L^hi^CD25^-^GFP^-^ naïve T cells from Tg4.WT mice were used as T responder cells for *in vitro* suppression assays, for donor cells in MBP-induced EAE, and as starting populations for induced Treg (iTreg) generation as previously described (22). Naïve populations from non-Tg4 mice were also used for iTreg generation, with stimulation provided by anti-CD3 (clone 145.2C11; eBioscience, Hatfield, UK) plus anti-CD28 (clone 37.51; eBioscience). For Th1 polarization, naïve T cells were cultured for 72 h on anti-CD3 plus anti-CD28 coated 24-well plates at a density of 0.25 × 10^6^/well in the presence of 10 U/ml rIL-2, 25 ng/ml rIL-12, and 25 ng/ml rIL-18 (all RnD Systems). After 48 h, cells were split and the concentration of IL-2 was raised to 20 U/ml. For suppression assays, Th1 cells were generated from Tg4 splenocytes with 10 μg/ml Ac1-9 in the presence of IL-12, IL-18, and IL-2 as described above. At the end of culture, cells were rested in 20 U/ml IL-2 for 48 h before use in the suppression assay.

### Induction of EAE

For MOG-induced EAE, C57BL/6, cKO, or cHet mice were immunized s.c. with 100 μg pMOG emulsified in CFA containing 200 μg heat-killed *Mycobacterium tuberculosis* H37RA (Sigma-Aldrich, Poole, UK). For MBP-induced EAE, C57BL/6xB10.PL host mice first received an i.v. injection of 1 × 10^6^ naïve Tg4 CD4^+^ T cells (with or without an equal number of Tg4 iTreg) 1 day before immunization with 10 μg Ac1–9 (4Tyr) in CFA. Mice also received 200 ng of pertussis toxin (Health Protection Agency, Dorset, UK) in 0.5 ml PBS i.p on the day of immunization and again 2 days later. Passive EAE was induced with MOG-responsive T cells using a previously described protocol (15). Clinical signs of EAE were assessed using the following scoring index: 0, no signs; 1, flaccid tail; 2, impaired righting reflex and/or gait; 3, partial hind limb paralysis; 4, total hind limb paralysis; 5, hind limb paralysis with partial front limb paralysis; 6, moribund or dead. Assessment of CNS (brain and spinal cord) immune cells was conducted as described previously (13).

### Tg4 *in vivo* suppression assay

C57BL/6xB10.PL mice were immunized with MBP peptide as described above for EAE induction 1 day after transfer of 1 × 10^6^ naïve Tg4 (CD90.1) responder cells with or without 1 × 10^6^ Tg4.WT or Tg4.T-bet^-/-^ iTreg. Seven days after immunization, spleens and draining lymph nodes (dLN) were isolated for assessment of responder cell accumulation, cell cycle status, and IFN-γ production by FACS as described below.

### T-cell transfer model of colitis

RAG1^-/-^ mice were injected i.v. with PBS or 5 × 10^5^ naïve WT CD4^+^ T cells (CD4^+^CD62L^hi^YFP^-^) from Foxp3^tm4(YFP/cre)Ayr^ mice in the presence or absence of 1.5 × 10^5^ KO nTreg (CD4^+^CD62L^hi^YFP^+^) from T-bet cKO mice. Mice were monitored daily and weighed three times a week until cull at 6 weeks after transfer. Colons were weighed and lymphoid cells isolated from the lamina propria. Briefly, the intestinal epithelial layer was removed by incubation in HBSS 2 mM EDTA for 30 min, and the remaining tissue digested with 1.25 mg/ml collagenase-4 (Worthington) and 30 μg/ml DNase-1 (Roche) in culture medium and disaggregated with a gentle MACS dissociator (Miltenyi). Retrieved lymphoid cells were stained and analyzed by flow cytometry.

### *In vitro* suppression and recall cytokine production assays

Suppression assays were performed by culturing naïve or Th1 Tg4 responder cells (1–2 × 10^4^/well) for 9 6h with increasing numbers of Tg4 iTreg or nTreg in the presence of 1 × 10^5^ irradiated (30 Gy) splenic C57BL/6xB10.PL APC and 10 μM MBP Ac1-9 peptide. 0.5 μCi [3H] thymidine (Amersham Biosciences, Amersham, UK) was added for the final 16 h and incorporation measured by β-scintillation counting (Wallac, Turku, Finland). For assays using Th1 responder cells, IFN-γ production was measured by ELISA. For assessment of recall cytokine production from immunized or EAE mice, lymphoid cells (splenocytes or dLN) were cultured in 96-well flat-bottom plates (BD) at 6 × 10^5^ dLN cells/well, 6 × 10^5^ CNS mononuclear cells/well, or 8 × 10^5^ splenocytes/well. Cells were stimulated with 20 μg/ml pMOG or 20 μg/ml MBP Ac1-9 for 24 h and cytokine production was measured by intracellular cytokine staining.

### Antibodies and FACS analysis

Cells were stained with the following antibodies and isotype controls (all from eBioscience, except where stated). Anti-CD4-eFluor450, anti-CD4-BV650 (BioLegend, San Diego, CA, USA), anti-CD90.1-APC, anti-CD45.1-FITC, anti-FoxP3-efluor450, anti-T-bet-PerCPCy5.5, anti-CXCR3-AF647, anti-CD25-APC, anti-CD62L-PE, anti-IFNγ-eFluor450, anti-GM-CSF-PE (BD), anti-IL-17-PerCPCy5.5, anti-CD11b-AF700 (BioLegend), anti-Ki-67-(PE/PerCPCy5.5), Rat IgG1 (PE, efluor450, PerCPCy5.5), Rat IgG2a (APC, efluor450, PerCPCy5.5, PE), mouse IgG1-AF647, and Armenian hamster IgG-APC. A fixable viability marker AF780 or eFluor455 (eBioscience) was also used. Flow cytometric data were acquired using a BD LSR Fortessa cell analyzer (BD) and data analyzed using FlowJo software (Treestar version 3.2.1, Ashland, OR, USA). Cell sorting used an ARIA II cell sorter (BD Biosciences). For intracellular cytokine staining in response to peptide, brefeldin A (eBioscience, 1000x stock) was added for the last 4 h of culture. Samples for transcription factor staining and samples for intracellular cytokine staining were washed once in FACS buffer (PBS, 2% FCS, 0.01% NaN_3_) and surface stained prior to processing for intracellular staining using proprietary buffers according to the manufacturer’s instructions (eBioscience for transcription factor staining or BD Pharmingen for cytokine staining). After incubation in fix/perm buffers, cells were stained for intracellular antigens.

### Statistics

Statistical analysis of results was performed using the Mann-Whitney *U* test or the Kruskal-Wallis with Dunn’s *post hoc* test.

## Results

### *Tbx*21^fl^/^fl^Foxp3-Cre conditional KO mice retain normal effector T-cell function

**Figure 1:**
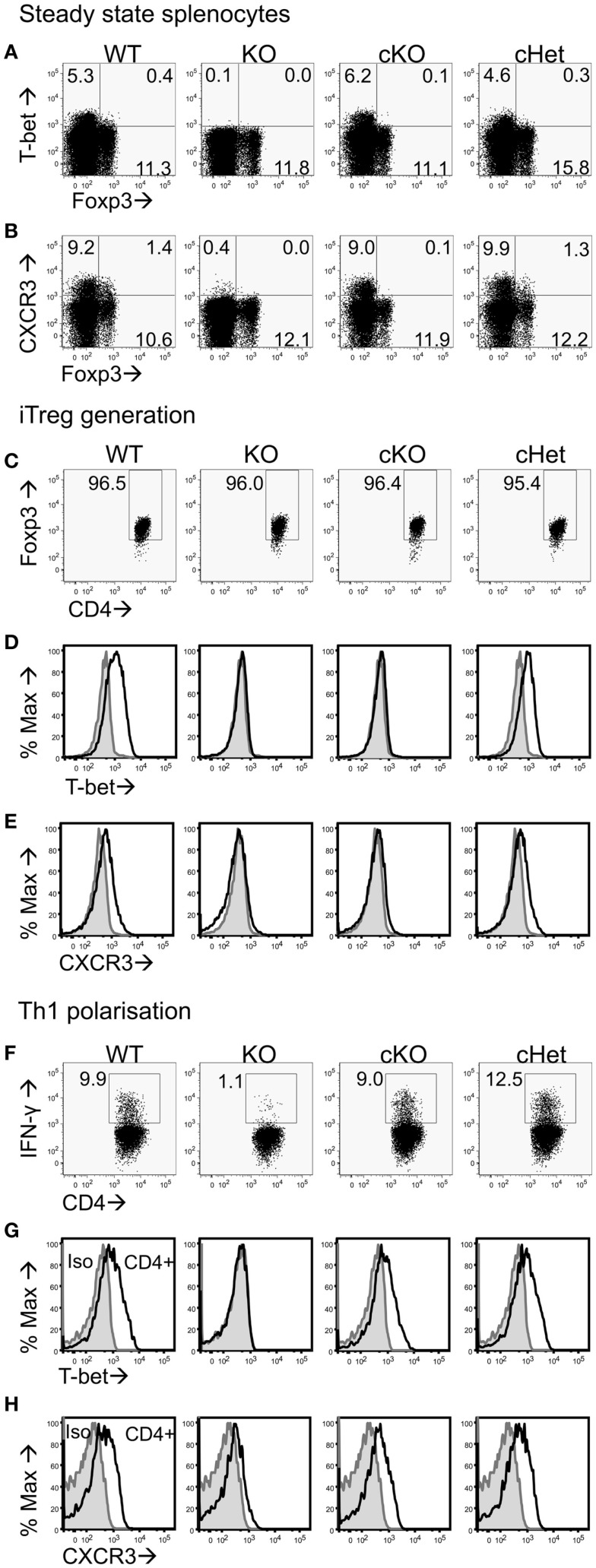
**Characterization of CD4^+^ T cells from T-bet cKO mice**. Representative plots of **(A)** T-bet vs. Foxp3 and **(B)** CXCR3 vs. Foxp3 staining within the CD4^+^ splenocyte population of unmanipulated C57BL/6 (WT), *Tbx21^-/-^* (KO), T-bet cKO, and T-bet cHet mice. Naïve CD4^+^ T cells were isolated from WT, KO, cKO, and cHet mice and cultured under iTreg generating **(C–E)** or Th1 polarizing **(F–H)** conditions. Representative plots of **(C)** Foxp3, **(D)** T-bet, and **(E)** CXCR3 staining within the CD4^+^ population following iTreg generation. **(F)** IFN-γ, **(G)** T-bet, and **(H)** CXCR3 staining within the CD4^+^ population of Th1 polarized cells. Data are representative of three experiments giving consistent results (*n* = 4 mice per group).

To test if T-bet is required by Treg to inhibit EAE pathology driven by T-bet sufficient T cells, we generated *Tbx*21^fl/fl^Foxp3-Cre cKO mice that specifically lacked T-bet expression only in the Foxp3-expressing Treg compartment (Figure 1A). As T-bet enables CXCR3 expression by Treg (7), Treg from these mice also showed abolished capacity for CXCR3 expression (Figure 1B). No differences were observed in T-bet or CXCR3 expression between the control T-bet cHet WT mice. Importantly, CD4^+^Foxp3^-^ cells from cKO mice expressed similar levels of T-bet and CXCR3 as those from WT and cHet mice (Figures 1A,B), indicating no impairment of T-bet expression within the Teff population.

We have previously reported that generation of Foxp3^+^ iTreg cells from naïve CD4^+^ T cells results in T-bet expression (22). To test whether cells from cKO mice lose the ability to express T-bet and CXCR3 upon induction of Foxp3 expression, we generated iTreg from naïve CD4^+^ T cells isolated from cKO and control mice. At the end of culture, >95% of the cells in all groups were expressing Foxp3 (Figure 1C), whereas T-bet and CXCR3 expression was only evident in the cells from WT and cHet mice (Figures 1D,E). Neither iTreg generated from T-bet KO mice nor cKO mice expressed T-bet or CXCR3, demonstrating the ability to express T-bet is lost upon Foxp3 expression in cKO mice.

In order to verify that Teff cells from cKO mice did not lack the ability to express T-bet, naïve CD4^+^ T cells were isolated and cultured under Th1 polarizing conditions. In contrast to Th1 cells generated from global T-bet KO mice, Th1 cells generated from cKO mice expressed IFN-γ, T-bet, and CXCR3 at levels similar to those seen in similar Th1 cells from either WT and control cHet mice (Figures 1F–H). The inability to express T-bet in cKO mice was therefore confined to CD4^+^Foxp3^+^ cells.

### Treg expression of T-bet is redundant for the resolution of EAE

**Figure 2:**
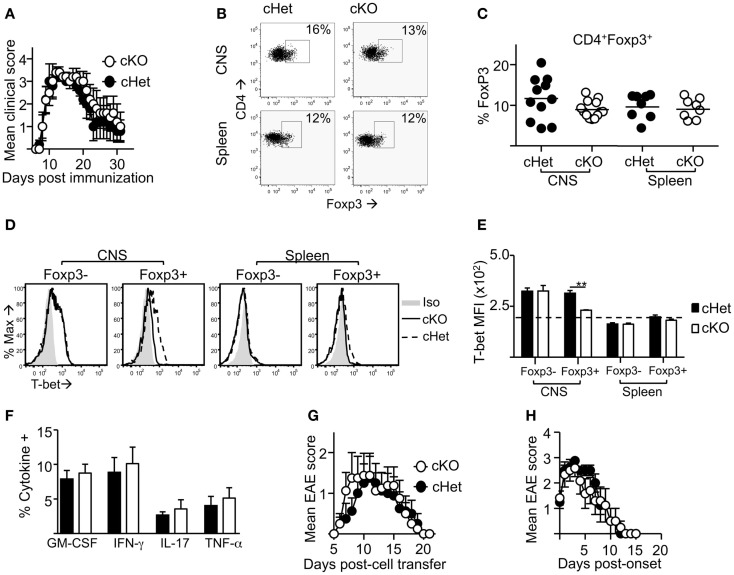
**Treg cells do not require T-bet for the resolution of EAE**. EAE was induced in T-bet cKO and T-bet cHet mice by immunization with pMOG. **(A)** Mean clinical scores ± SEM. Incidence in both groups was 100%. **(B,C)** CD4^+^ cells expressing Foxp3 within the CNS and spleen at day 13 of disease. **(D,E)** T-bet staining (MFI) within the CD4^+^Foxp3^-^ and CD4^+^Foxp3^+^ populations of the CNS and spleen **(E)** (dotted line represents MFI of isotype control staining), **(F)** GM-CSF, IFN-γ, IL-17, and TNF-α production following overnight culture of CNS mononuclear cells with pMOG (gated on CD4^+^CD11b^-^ T cells). Data are from one of three experiments giving consistent results, *n* = 8 mice per group. **(G)** Mean clinical EAE scores ± SEM of cKO or cHet mice that received passive transfer of 3 × 10^6^ WT IL-12-conditioned pMOG-reactive cells. Data are from one of two experiments giving consistent results, *n* = 8 mice per group. Maximum EAE score for each mouse was 0, 0, 0, 0, 3, 3, 3, 3 (cHet) and 0, 0, 2, 2, 3, 3, 3, 3 (cKO). **(H)** Mean EAE score ± SEM from day of onset of those mice that developed disease after passive transfer.

Following induction of EAE by active immunization with pMOG, there was no difference in the severity or resolution of disease between cKO and cHet control mice (Figure 2A). Accordingly, no differences were observed in the proportion (Figure 2B) or number (data not shown) of Foxp3^+^ cells recruited into the CNS during disease (Figures 2B,C), suggesting no deficit in the ability of Treg to migrate into the CNS of cKO mice. In line with our previous report (16), T-bet was expressed by Treg within the CNS and not the periphery of cHet mice (Figures 2D,E), verifying that this phenotype is enriched at the site of inflammation in mice where T-bet expression is unrestricted. Despite the lack of T-bet expression in the CD4^+^Foxp3^+^ population of the cKO mice (Figures 2D,E), there was also no difference in the proportion of CD4^+^ cells producing pro-inflammatory cytokines, including IFN-γ, within the CNS (Figure 2F). Of note, no differences were observed in the proportion of T cells staining for GM-CSF, the only T-cell-derived pro-inflammatory cytokine that is believed to be critical for EAE pathogenesis (23, 24). Although IFN-γ producing CD4^+^ T cells are evident within the CNS of this model, the induction of EAE through immunization with myelin-derived peptides results in a heterogeneous pathogenic T-cell population including both Th1 and Th17 cells (14, 15); indeed, IL-17 producing CD4^+^ T cells were evident in the CNS (Figure 2F). To better scrutinize the requirement of T-bet expression in Treg for the control of Th1 responses during EAE, disease was induced via transfer of IL-12-conditioned antigen-experienced WT T cells into cKO or cHet control mice. Similarly to active EAE, the disease course was no different between cKO and control cHet mice (Figure 2G) and no disparity was observed in the resolution of clinical signs (Figure 2H) demonstrating that Treg do not require T-bet expression for the resolution of Th1-driven CNS inflammation.

### T-bet-deficient Treg can suppress Th1 responses both *in vitro* and *in vivo*

**Figure 3:**
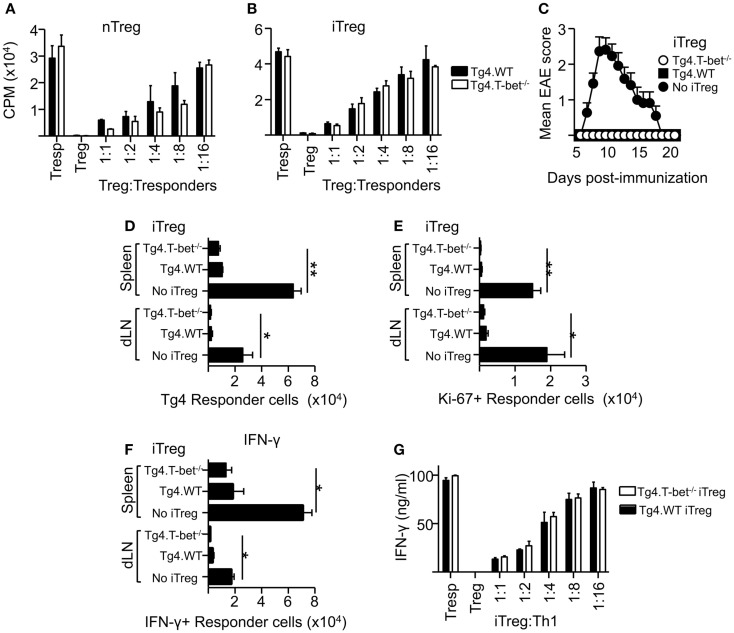
**T-bet is not required for Treg cell suppressive function**. Varied numbers of Tg4.WT or Tg4.T-bet^-/-^ nTreg **(A)** or iTreg **(B)** were co-cultured with a fixed number of naïve Tg4 responder cells and stimulated with MBP Ac1-9 (as described in Section “Materials and Methods”). Data are from one of two experiments giving consistent results. **(C–F)** C57BL/6xB10.PL were immunized with MBP Ac1-9 1 day after transfer of 1 × 10^6^ naïve CD4^+^ Tg4 responder cells (CD90.1), with or without 1 × 10^6^ Tg4.WT or Tg4.T-bet^-/-^ iTreg (CD45.1) for development of EAE **(C)** (mean clinical scores ± SEM), or for *ex vivo* analysis of spleen and dLN after 7 days **(D–F)**. *Ex vivo* numbers of Tg4.CD90.1 responder cells **(D)**, Ki-67^+^ responder cells **(E)**. IFN-γ^+^ responder cells after overnight culture with MBP peptide **(F)**. Data are from one of three experiments giving consistent results, *n* = 5 mice per group. **(G)** IFN-γ production by Tg4 Th1 responder cells co-cultured with Tg4.WT or Tg4.T-bet^-/-^ iTreg cells. Data are from one of two experiments giving consistent results.

As a lack of T-bet expression did not appear to inhibit the resolution of active EAE or passive EAE using IL-12-conditioned T cells, the ability of T-bet-deficient Treg to suppress the proliferation of naïve CD4^+^ T cells was tested. These experiments used MBP-responsive Treg derived from the previously described Tg4xFoxp3.LuciDTR-4 mice (22) (here referred to as Tg4.WT) and compared their function with Treg from *Tbx21^-/-^*Tg4xFoxp3.LuciDTR-4 mice (Tg4.T-bet^-/-^). T cells from both of these strains express a transgenic TCR specific for the MBP (Ac1-9) peptide and eGFP under the Foxp3 promoter, enabling high purity Foxp3^+^ nTreg and Foxp3^-^ naïve T-cell populations to be isolated by FACS. *In vitro* assays testing the ability of these two Treg populations to inhibit the proliferation of naïve CD4^+^ Tg4.WT responder T cells showed that their suppressive abilities were equivalent. This was the case using either freshly isolated nTreg or *in vitro* generated iTreg (Figures 3A,B). The same was observed for polyclonal T-bet-deficient nTreg and iTreg and polyclonal naïve T cells following stimulation with anti-CD3 (data not shown).

To test suppressive function *in vivo*, we used a previously described system in which naïve CD90.1-expressing Tg4 responder cells, with or without a corresponding cohort of CD45.1-expressing Tg4 iTreg, were transferred into B10.PLxC57BL/6 host mice (expressing CD45.2 and CD90.2) prior to immunization with MBP peptide in CFA (22). Tg4.T-bet^-/-^ iTreg were able to fully inhibit the resulting development of EAE (Figure 3C). Consistent with this, accumulation of Tg4 responder cells in the spleen and dLN was significantly reduced by co-transfer of either Tg4.WT or Tg4.T-bet^-/-^ iTreg cells (Figure 3D). Similar results were obtained when using nTreg cells (data not shown). In accordance with this, the numbers of responder Tg4 cells in cell cycle (as determined by *ex vivo* Ki-67 staining) and staining for IFN-γ after *in vitro* restimulation with MBP peptide were decreased in the spleen and dLN of mice that had received either WT or T-bet^-/-^ iTreg cells (Figures 3E,F).

To test the ability of T-bet^-/-^ Treg to suppress pre-activated Th1 cells, an *in vitro* suppression assay was conducted using Tg4 Th1 cells as responder cells. Tg4.WT and Tg4.T-bet^-/-^ iTreg suppressed production of IFN-γ by the responder Th1 cells with equivalent efficacy as determined by iTreg cell dilution (Figure 3G). From these data, we conclude that T-bet^-/-^ Treg are effective at both preventing and inhibiting antigen-driven Th1 responses.

### T-bet-deficient Treg can suppress colitis

**Figure 4:**
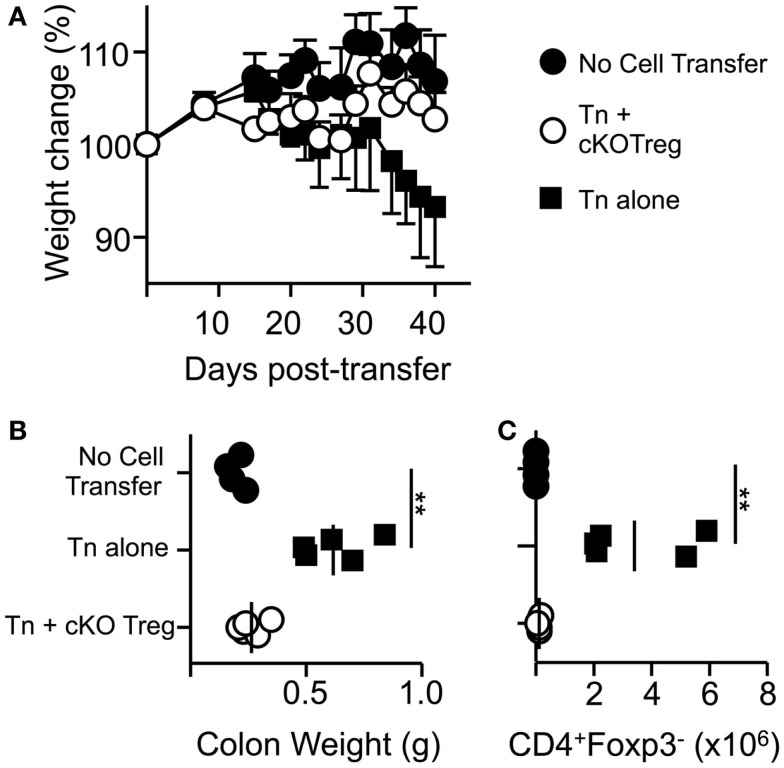
**Treg cells do not require T-bet to suppress T-cell-driven colitis**. RAG1^-/-^ mice received PBS, 5 × 10^5^ naïve WT CD4^+^CD62L^hi^YFP^-^ T cells alone, or in combination with 1.5 × 10^5^ T-bet cKO CD4^+^CD62L^hi^YFP^+^ T cells i.v. and were monitored for weight loss for 6 weeks. **(A)** Mean body weight change ± SEM. At the end of the experiment, by colon weight was measured **(B)** and numbers of CD4^+^Foxp3^-^ T cells present in the lamina propria were determined by flow cytometry **(C)**. Data are from one of two experiments giving consistent results (*n* = 4–5 mice per group).

In order to determine if T-bet is required by Treg to suppress another model of T-cell-driven inflammation, a T-cell transfer model of colitis similar to those previously described (25) was used in which pathogenesis can be prevented by co-transfer of Treg cells. Transfer of naïve WT T cells alone into RAG1^-/-^ host mice led to a loss in total body weight and an increase in colonic weight (Figures 4A,B). Co-transfer of Treg isolated from T-bet cKO mice allowed maintained body weight and prevented changes to colon weight. Furthermore, CD4^+^Foxp3^+^ cells were present and numbers of CD4^+^ Foxp3^-^ cells were significantly reduced within the lamina propria of mice that received T-bet cKO Treg compared to those that received naïve T cells alone (Figure 4C). We conclude that Treg do not require T-bet expression to suppress the T-cell transfer model of colitis.

## Discussion

Unaltered Treg infiltration of the CNS and EAE resolution in cKO mice suggests that, in this disease setting, expression of T-bet and CXCR3 by CNS-targeting Treg may be an epiphenomenal consequence of exposure to the local inflammatory milieu rather than a necessity for their function.

Previous studies have implicated T-bet and CXCR3 as important factors in the suppression of Th1 responses by Treg, either by endowing Treg with superior homeostatic properties or by facilitating their migration to, and accumulation at, sites of Th1 inflammation (7, 10, 18). However, several other studies have reported that T-bet-deficient Treg are equally suppressive as WT Treg *in vitro* (26, 27), and in certain Th1-mediated diseases can even have greater suppressive capacities than WT Treg (28). Together, these reports imply that T-bet-deficient Treg may have some decreased ability to traffic to sites of Th1-mediated inflammation but have no defect in their suppressive modalities.

The requirement for CXCR3 expression by Treg cells (and indeed Teff cells) to allow their migration into the CNS during EAE is of some debate. It has been reported that blockade of CXCR3 can inhibit leukocyte migration into the CNS and the induction of passive EAE, but has no effect in the active EAE model (29). Inhibition of the CXCR3 ligand CXCL10 has also been reported to inhibit disease induction and T-cell migration (30, 31). However, other studies have shown increased disease severity in CXCR3-deficient mice, with one report detecting no difference in overall leukocyte migration (17), and another demonstrating reduced accumulation of Treg within the CNS (18). Despite these observations, the exact role of CXCR3 expression on Treg cells during EAE has not previously been investigated. Through the use of global T-bet KO mice, we have previously observed that T-bet, and hence CXCR3, is not required for the induction of EAE, nor for the resolution of disease in this model (32). Furthermore, we have not observed differences the recruitment of Treg into the CNS during EAE in T-bet KO mice compared to WT mice (data not shown). However, the dominant cytokine produced by CD4^+^ Teff cells in the CNS of T-bet KO mice is IL-17 (32), rather than IFN-γ as seen in WT mice. This may indicate that, rather than T-bet and CXCR3 expression, Treg would require a Th17-like phenotype in order to resolve EAE in this model. Through the use of T-bet cKO mice, we have demonstrated that, despite a normal CNS Teff cell compartment dominated by IFN-γ^+^ cells, T-bet-deficient Treg remain able to migrate to the site of inflammation and coordinate disease resolution. Therefore, specialization may not be universally required for Treg migratory potential or function.

Few studies have investigated the ability of T-bet-deficient Treg to specifically suppress Th1 responses. Koch et al. demonstrated that T-bet-deficient Treg were unable to protect *scurfy* mice from spontaneous autoimmune inflammation and subsequent death (7). Similarly to EAE, the inflammation observed in *scurfy* mice is heterogeneous in nature where both Th1 and Th17 cells can be detected (7, 33), although Th1 responses are thought to dominate both models. In accordance with this, Treg cells transferred into *scurfy* mice up-regulate expression of T-bet and CXCR3, as we see in the CNS of EAE mice. Despite these similarities, T-bet appears necessary for Treg to control the Th1 response in multi-organ inflammation observed in *scurfy* mice but not CNS inflammation during EAE. This might be accounted for by different requirements for migration to distinct sites of inflammation, or by variation in the inflammatory milieu between models. However, disease can occur in either model in the absence of IFN-γ or T-bet (and therefore CXCR3) (32–35) indicating other pathological roles for inflammatory T cell other than Th1 cells. Indeed, the study by Koch et al. did not report on survival rates between *scurfy* mice that did not receive Treg compared to those that received T-bet-deficient Treg, leaving open the possibility that T-bet-deficient Treg did have a partially beneficial role in that model.

We also used a passive transfer EAE model (transferring IL-12-conditioned Teff cells). It is important to note that T-bet KO Teff cells cannot transfer EAE in this model (32). However, we demonstrate here that host Treg cells do not require T-bet to resolve this T-bet-dependent pathology. The lack of requirement for T-bet expression for Treg function was not a feature restricted to CNS inflammation, as it was replicated in a colitis model also reported to require Th1 responses (28, 36). Consistent with this, we demonstrated that T-bet-deficient Treg were capable of suppressing antigen-driven Th1 function (IFN-γ production) both *in vitro* and *in vivo*.

In summary, T-bet is not required by Treg cells for their suppression of Th1 responses, or for their migration into the CNS to coordinate the resolution of EAE. Despite the phenotypic changes seen in WT Treg cells during EAE (gain of T-bet and CXCR3 expression), these characteristics appear to play no role in their function. These results suggest that the manipulation of Treg cells to drive T-bet and CXCR3 expression would not endow them with a therapeutic advantage.

## Conflict of Interest Statement

The authors declare that the research was conducted in the absence of any commercial or financial relationships that could be construed as a potential conflict of interest.
